# Idiopathic toe walking may impact on quality of life

**DOI:** 10.1186/1757-1146-8-S2-O40

**Published:** 2015-09-22

**Authors:** Cylie Williams, Terry Haines

**Affiliations:** 1 Physiotherapy Department, Monash University, Peninsula Campus, Vic, Australia; 2 Allied Health Research Unit, Southern Health, Vic, Australia

**Keywords:** Quality of life, idiopathic toe walking

## Background

Idiopathic toe walking (ITW) is a diagnosis made in the absence of a medical condition known to cause toe walking gait. ITW has been associated with mild speech and motor delays and a reduction in ankle range of motion. It is not known know if ITW impacts on the quality of life.

## Methods

This research was a cross-sectional online survey. Parents of children aged 5-17 and people above 8 years, who previously or currently have an ITW gait, were recruited. Recruitment was through public and private health services and online parenting forums. Questions about known diagnoses, birth and developmental history were used for screening to reduce the chance of the toe walking being related to a medical condition. Data collected included PedsQL 4.0 Generic Core Scale via either parent proxy or child/adult report, the parent proxy PedsQL Family Impact module, past treatments of ITW and any current localised musculoskeletal pain. The results of the PedsQL 4.0 were compared to healthy population, cerebral palsy and Attention and Hyperactivity Disorder (ADHD) data for the parent proxy and child reports only.

## Results

There were 141 responses and following screening, 82 responses were included for analysis. The parent proxy responses (n=49) determined a lower Total (p<0.01), Psychosocial (p<0.05) and Emotional subscale (p<0.01) scores on the PedsQL 4.0 compared to the healthy group, but higher subscale scores than the cerebral palsy and ADHD groups (p<0.01). There was no identified impact of ITW on the family. In the child self report (n=8), the ITW group scored lower in the Total, Physical, Psychosocial and School scores (p<0.01) than the healthy population only. Adults (n=25) who toe walked in the past or currently toe walked reported continued pain at the back of their calves with everyday activity and increased corns and callus at the balls of their feet.

## Conclusion

ITW gait may impact on the quality of life in children and adults who exhibit this gait type. Podiatrists should be aware of this potential impact when assessing and treating this gait type.

